# Translation and Psychometric Properties of the Portuguese Version of the Timed Instrumental Activities of Daily Living (TIADL)

**DOI:** 10.3390/geriatrics8060124

**Published:** 2023-12-18

**Authors:** Luis Galhardas, Armando Raimundo, José Marmeleira

**Affiliations:** 1 Departamento de Desporto e Saúde, Escola de Saúde e Desenvolvimento Humano, Universidade de Évora, Largo dos Colegiais, 7000-727 Évora, Portugal; ammr@uevora.pt (A.R.); jmarmel@uevora.pt (J.M.); 2 Comprehensive Health Research Centre (CHRC), Palácio do Vimioso, Gabinete 256, Largo Marquês de Marialva, Apart. 94, 7002-554 Évora, Portugal

**Keywords:** reliability, nursing homes, instrumental activities, performance accuracy

## Abstract

(1) Background: to examine the psychometric properties of the Portuguese version of the Timed Instrumental Activities of Daily Living (TIADL) in nursing home residents. (2) Methods: Fifty-two participants (85.8 ± 4.2 years) were assessed on two occasions, 10–14 days apart. The same rater administered all assessments. Internal consistency was analysed through Cronbach’s α. The reliability was estimated using the intraclass correlation coefficients (ICCs), and the standard error of the mean (SEM) was used to estimate the minimal detectable change (MDC). Construct validity was determined by Spearman’s correlation coefficients. (3) Results: For internal consistency, Cronbach’s α (0.81) revealed high internal reliability. All of the subtests demonstrated good or excellent reliability and also presented acceptable measurement precision, considering the criterion SEM < SD/2. According to Spearman’s rho, correlations with the Portuguese version of the TIADL, the Useful Field of View test, and semantic and phonemic fluency tests were significant, with moderate positive and negative correlations (0.4 < *r_s_* < 0.69). (4) Conclusions: The Portuguese version of the TIADL had good to excellent test–retest reliability (ICC > 0.90) and acceptable measurement precision. This test could be a valuable clinical tool for assessing actual performance in instrumental activities of daily living in nursing home residents.

## 1. Introduction

The percentage and absolute number of older people are increasing sharply worldwide, and most people can expect to live over 70 years [1]. The ageing process leads to inevitable life changes and is characterised by a progressive decline in physiological and psychological functions [2,3,4]. Frequently, older adults experience limitations in aerobic capacity, muscular strength, and balance [5,6,7], and declines in cognitive capacities such as memory, attention, and information processing [5,8,9]. Age-related alterations lead to a decline in the performance of activities of daily living, which have a major impact on the autonomy and independence of this population [10,11]. 

Usually, assessment tools for IADLs evaluate the individual’s ability for specific actions (e.g., using the cell phone) [12,13], that is, they are often others’ subjective assessments, such as a psychologist, of a person’s ability to perform a given task. The technician, based on his observation and data collected on the person, assigns a score but does not evaluate the person’s effective performance at any time. In this type of performance accuracy-based assessment, the test score does not reflect the temporal aspects of execution and does not consider the well-known age-related slowing that may affect physical and cognitive functions and limit a person’s capacity for independence [14]. For instance, the level of performance of a given task is evidently different between a person who takes three minutes to complete it and a person who performs it in one minute.

In this article, we describe the translation process and the study of the psychometric properties of the Timed Instrumental Activities of Daily Living (TIADL) tasks [15], which is a performance-based assessment of IADLs. This assessment method analyses the speed at which several activities of daily living can be accomplished (in seconds). In its original version, the tasks involve five IADL domains (and five tasks), namely, communication, finances, cooking, shopping, and medicine. This method showed good test–retest reliability (Pearson correlation coefficient 0.85) [15].

To the best of our knowledge, there is no similar assessment method for the Portuguese population; therefore, the cultural adaptation and validation of the TIADL could fill a void in the objective assessment of the performance of activities of daily living by older adults. In the present study, we chose to focus on older adults living in nursing homes, a group of older adults which have grown markedly over the years, and for whom it is important to have suited instruments of assessment of their functional status. Hence, people living in long-term care facilities, such as nursing homes, tend to be frail [16,17,18] and, consequently, it is imperative to design and develop interventions that have a positive impact on their functional capacity. Consequently, the accurate assessment of nursing home residents’ capabilities is of extreme importance for health personnel to design proper (and individualised) intervention activities, monitor patient progress, and evaluate the effects of the interventions carried out [19]. 

The present study aims to translate and culturally adapt the original TIADL [15] into Portuguese and to assess its validity and reliability in older nursing home residents.

## 2. Materials and Methods

### 2.1. Sample

Seven nursing homes were selected by convenience in the region of Évora (Portugal). After obtaining approval to conduct our research in the institutions, 52 potential participants were identified and invited to join in the study with the assistance of the health care personnel. All residents who were invited agreed to participate in the study and their eligibility was confirmed according to the following inclusion criteria: being aged 75 years or older; living in a nursing home; being able to walk independently (with or without a walking aid); and present a normal cognitive status according to the Portuguese version of the Mini-Mental State Examination [20,21]. 

We obtained informed consent from all potential residents for joining the study. This study was approved by the Ethics Committee of the University of Évora and was carried out in accordance with the Declaration of Helsinki [22].

Table 1 shows the general characteristics of the participants. The sample included 36 women and 16 men aged 77–95 years. Considering the World Health Organization categorisations regarding body mass index (BMI), 46% of the participants were overweight (≥25 kg/m^2^) and 8% were obese (≥30 kg/m^2^). All participants attended school and had an average education of 4.7 years.

### 2.2. TIADL–Original Version 

The original TIADL was developed by Owsley et al. [15] to evaluate the actual performance (performance-based) of older adults in five common activities of daily living. Each task corresponds to an IADL domain, namely, communication, finances, cooking, shopping, and medicine. In the original study, the authors described the assessment protocol and included a set of instructions to be used for each TIADL task. Specific materials for all of the tasks were organised into a ‘kit’ to simplify portability and ease of administration [15]. In all activities, performance is measured by the time (in seconds) that the person takes to accomplish the task. 

The original study showed that the TIADL had good test–retest reliability (Pearson correlation coefficient 0.85), with the test and retest sessions occurring 7–8 weeks apart. For this analysis, the authors used data from 47 older adults (mean age 74 years), all readers, and living independently of formal care. The original study reported moderately significant associations (R^2^ 0.14–0.29) between the TIADL and the Useful Field of View (UFOV^®^), the Rey Auditory-Verbal Learning Test, and the Word Series test [15]. The TIADL has been used frequently [23,24,25].

### 2.3. Translation and Cultural Adaptation

The international guidelines of the World Health Organization [26] were considered to translate the original English TIADL [15] into Portuguese. The translation was performed considering relevant cross-cultural elements. One translator, a specialist in gerontology, translated the TIADL protocol from English to the Portuguese language (step 1). Then, two kinesiology specialists, bilingual in Portuguese and English languages, checked the translation for possible inconsistencies and made the necessary improvements (step 2). In the next step, a team of Portuguese researchers (three health professionals) examined the content and precision of the translated version to confirm the proper interpretation. After that, all content was adjusted according to all of the feedback from the researchers (step 3). A back translation was performed, where the Portuguese manuscript was translated back to English (original language) by an independent bilingual researcher without knowledge of the original English version of the TIADL (step 4). After comparing the original version and that obtained in step 4, a pre-final version was obtained. At this stage, we used a focus group to test the pre-final version (pre-testing and cognitive interviewing) [27]. The focus group included 10 older adults (five women) and allowed researchers to implement minor modifications to the instrument. After all of these procedures, the final version of the Portuguese TIADL was completed (Supplementary Materials).

### 2.4. TIADL Tasks

The original TIADL includes five tasks [15], involving five IADL domains. Below we present these tasks and our adaptations:

- Task 1—“Communication”. This task requires searching for a specific telephone number in a phone book (residential phone directory).
- Task 2—“Finances”. The participant is required to make a money change with real coins (euros in our study).
- Task 3—“Food”. The participant should read the first three ingredients on a can of food (on three different cans).
- Task 4—“Shopping”. The participant is required to find two food items on a simulated shelf. For this task, it was necessary to construct a simulated shelf of assorted food items. For our study, a shelf was built based on and with an appearance like that used in the original protocol.
- Task 5—“Medicine”. The participant read the instructions on two medicine containers. The original protocol used two medicine containers with the real prescription label attached. In our study, we used two different patient information leaflets.

The score for this assessment method is the time (in seconds) it takes the person to complete the tasks. The examiner uses a digital stopwatch to record the time spent. 

### 2.5. Data Collection

This was a test–retest reliability study with all outcome measures collected on two occasions (10 to 14 days apart). The same kinesiologist administered all of the tests for this study at both time points. In the process of data acquisition and results dissemination, we employed the COSMIN taxonomy of measurement properties [28].

#### 2.5.1. Reliability

Before collecting the data, the kinesiologist became familiar with the protocol and administered it to five older adults in nursing homes under the supervision of a senior researcher with experience in this field. Also, about one week before the initial assessment, all nursing home residents involved in this study participated in a training session to become familiar with the methods to control for learning effects. All tests were performed individually and in a quiet room at the nursing homes.

We collected data for test–retest reliability, measurement error, and internal consistency.

#### 2.5.2. Correlative Measures for Validity

For construct validity, we analysed the correlation of the Portuguese TIADL with other assessments of processing speed, namely, the Useful Field of View test (UFOV^®^; Visual Awareness, Inc., Chicago, IL, USA) [29,30] and the phonemic and semantic verbal fluency tests [31]. 

Useful Field of View (UFOV). The UFOV test was also used in the original study of the TIADL [15] for assessing construct validity. This test is related to everyday performance measures [32,33], and improvements in the UFOV test have been associated with better-quality performance in everyday capabilities [33,34]. In the current study, we used Subtest 1 (processing speed) and Subtest 2 (divided attention). The score was the time (in milliseconds) taken from when the items appeared on the participants’ computer screen to when the participant correctly named it in 75% of the trials [35].

Semantic and phonemic fluency. According to the authors of the original version of the TIADL [15], this assessment method is related (crudely) to memory and reasoning and depends on processing speed. We chose the semantic and phonemic fluency tests [31] to correlate with the TIADL. The semantic and phonemic fluency tests are simple and brief assessment tools that measure nonmotor processing speed, executive functions, and language production [31]. In the semantic fluency test, participants are required to orally name as many animals as possible within one minute. Regarding phonemic fluency, the participants are encouraged to name as many words as possible that began with a specific letter (excluding proper nouns). The test consisted of three one-minute trials.

### 2.6. Statistical Analysis

Statistical tests were used to examine the internal consistency, reliability, and validity of the Portuguese version of the TIADL. The internal consistency of the Portuguese version of the TIADL test was estimated by Cronbach’s α coefficient using the criteria for an instrument with few items (less than 10); values of Cronbach’s α above 0.9 indicate excellent internal reliability, values between 0.7 and 0.9 indicate high internal reliability, values between 0.5 and 0.7 indicate moderate internal reliability, and values below 0.5 indicate low internal reliability [36].

Reliability refers to the level of association (correlation) of repeated measurements [37,38]. The test–retest reliability was assessed with the intraclass correlation coefficients (ICCs), applying the two-way mixed-effects model analysis of variance [37,38]. An ICC value larger than 0.90 indicates excellent reliability, a value of 0.75–0.90 indicates good reliability, a value of 0.50–0.75 indicates moderate reliability, and a value of <0.50 indicates poor reliability [39]. As well, the reliability depends on the variability of the scores from trial to trial (within-subjects/measurement), and this value is not a sample-dependent quantity since the range of individual scores is not considered [38]. The reliability of the Portuguese version of the TIADL was studied through the standard error of the mean (SEM) to estimate the minimal detectable change (MDC) at the 95% confidence level. The formulas used to examine the SEM and MDC were SEM = SD × √(1-ICC) and MDC95 = SEM × √2 × 1.96, respectively. SD corresponds to the average of standard deviations from test and retest moments [38]. For the SEM, we considered the criterion SEM < SD/2 for acceptable or nonacceptable measurement precision [38,40,41]. MDC95 refers to the smallest change that must occur to be considered a real outcome of an intervention or exercise program [38,42]. We report the SEM and MDC95 data in the same unit of assessment as the corresponding assessment protocols. 

Additionally, we computed Spearman’s correlation coefficients to assess the correlations of the Portuguese version of the TIADL with the UFOV test, semantic fluency tests, and phonemic fluency tests, specifically evaluating construct validity; values less than 0.4 represent a weak correlation, values from 0.4 to 0.69 represent a moderate correlation, and values from 0.70 to 0.99 have a strong correlation [43]. In this correlation analysis, we applied the mean Z-score of the TIADL test since it captures the overall performance. Z-scores represent a statistical measurement of a score’s relationship to the mean in a group of scores.

Finally, to identify possible systematic bias between assessment moments, after checking for data normality with the Shapiro–Wilk test, we performed the paired sample *t*-test or the Wilcoxon signed-ranks test. The level of significance was established to be *p* < 0.05. All data were analysed using the SPSS statistical program (version 20.0, Inc., Chicago, IL, USA).

## 3. Results

### 3.1. Psychometric Properties of the Portuguese TIADL

#### 3.1.1. Internal Consistency

The Cronbach’s α coefficient test results were 0.81 in the five tasks of the Portuguese TIADL, indicating high internal reliability.

#### 3.1.2. Test–Retest Reliability and Measurement Error

Table 2 shows the mean scores and standard deviations of the subtests and their reliability values. Regarding the intraclass correlation coefficient, all of the subtests showed good (ICC > 0.75) or excellent (ICC > 0.90) reliability.

Regarding the measurement error, all tasks presented acceptable measurement precision, according to the criterion SEM < SD/2 for acceptable or nonacceptable measurement precision [38,40,41].

The scores obtained in test and retest sessions were very similar for all components, and the paired sample t-test or the Wilcoxon signed-rank test confirmed that they were not significantly different (*p* < 0.05). Hence, the mean difference in the tasks was 4.37 s (3.66%; *t* = 0.88, *p* = 0.17) for communication, −0.07 s (−0.21%; *w* = −0.21, *p* = 0.83) for finances, −1.47 s (−2.75%; *w* = −0.07, *p* = 0.94) for purchases, −3.93 s (−3.48%; *w* = −1.88, *p* = 0.06) for food, and 0.27s (0.53%; *t* = 0.89, *p* = 0.77) for medicine.

As previously described, the MDC_95_ refers to the minimum variation that must exist to be considered a real effect of an intervention [38,42]. For this reason, we present the values for future use, allowing professionals to consider these data. We do not find any systematic bias in these data.

#### 3.1.3. Construct Validity

The results for Spearman’s correlation coefficients regarding the correlations of the Portuguese TIADL test with the UFOV test and semantic and phonemic fluency tests are shown in Table 3. All values were moderately associated, with values from 0.47–0.58 (*p* < 0.01).

## 4. Discussion

In Portugal, assessment methods and protocols to assess performance (without self-report) for instrumental activities of daily living in older adults, especially those that live in residential care facilities, are lacking. Based on this, we aimed to translate, culturally adapt, and study the psychometric properties of the TIADL [15], creating the Portuguese version of the TIADL.

For examining the psychometric properties, we analysed the internal consistency, test–retest reliability, and construct validity. Regarding internal consistency, our results suggested that the Portuguese TIADL exhibited acceptable values (Cronbach’s α of 0.81), indicating high internal consistency [36,44]. Unfortunately, the original TIADL article [15] did not report values of internal consistency, and we have not been able to find data on the internal consistency of the TIADL or other similar assessment methods. The Cronbach’s α values that we found are considered acceptable, with high internal consistency, because it is an assessment method with few items. Other recent studies applied this same criterion [45,46]. However, even applying the most classic criterion for the interpretation of Cronbach’s α values, the 0.81 obtained in our study indicates a very good level of reliability. A generally accepted rule is that an α of 0.6–0.7 indicates an acceptable level of reliability, and 0.8 or larger indicates a very good level [47].

Similar to findings from the original TIADL [15], the test–retest reliability results indicate that the Portuguese TIADL also shows good to excellent reliability (ICCs from 0.90 to 0.95). As described before, all of the subtests present acceptable measurement precision, according to the criterion SEM < SD/2. These values are very useful for future implementation at the clinical level.

Construct validity is the extent to which the measurements used really test the hypothesis or premise that they are assessing [48]. In the current study, we investigated convergent construct validity, which assesses the relationship between the construct in similar assessment methods. This type of validity arises when the method measures concepts similar to that of other instruments [49]. To examine the convergent construct validity of the Portuguese TIADL, we selected the UFOV test (Visual Awareness, Inc.) and the semantic and phonemic fluency tests [31] as the validation tools. As we described before, the UFOV test was used in the validation of the original TIADL protocol [15], thus reinforcing the relevance of using this method in our work. The results obtained show (positive and negative) moderate correlations between the Portuguese version of the TIADL and the two methods selected for examining convergent construct validity. In the original study of the TIADL [15], the authors obtained significant crude associations (linear regression) between TIADL scores and the UFOV test. Such findings are in accordance with what we find in our data: older people who perform better on the UFOV test tend to be better at instrumental activities of daily living assessed with the TIADL. 

Considering that the UFOV test directly evaluates components such as processing speed, several authors [33,50,51] indicated that if these capacities are better, then the capacity to perform instrumental activities of daily life will be better. This strengthens the data obtained in our study. People with better performance on the UFOV test tended to present a better performance in the Portuguese version of the TIADL. 

To assess the processing speed, we also selected the semantic and phonemic fluency tests [31] that, like the TIADL, also directly involve processing speed [31,52]. Both tests (semantic and phonemic) showed moderate negative correlations with the Portuguese TIADL (Table 3). In general, people with better fluency (more words) tend to be better at the TIADL (less time). These findings are supported by the processing speed theory [53], which indicates that increasing age is associated with a slower speed, which is reflected in the performance of different activities that depend on processing speed. For assessing memory, reasoning, and speed of processing in the original protocol [15], the Rey Auditory-Verbal Learning Test [54] was used. The data obtained showed that the deficient performance on each cognitive measure was crudely associated with more time to complete tasks on the TIADL. These data also reinforce those obtained by us.

This study had some limitations. First, the sample included more older women than men, although it should be noted that in Portugal, in nursing homes, there are more women than men, especially at advanced ages (as is the case of participants in the present study). Second, we did not examine the level of frailty of the participants nor their ability to perform activities of daily living. Third, the visual capabilities of the participants were not controlled. Fourth, the examination of responsiveness and criterion validity was not conducted in this study; hence, future investigations should undertake this analysis. Fifth, the sample was based on convenience and was relatively small; future studies should employ randomized samples with a larger number of participants. Sixth, since our sample exclusively included individuals without cognitive impairment, these findings cannot be extrapolated to all nursing home residents. Subsequent studies should also incorporate participants with cognitive impairment.

There are some important strengths of this study. The Portuguese cultural adaptation of the TIADL showed very satisfactory reliability in older adults living in nursing homes. As previously mentioned, most assessment methods evaluate indirectly the IADL (e.g., questionnaire), whilst the TIADL evaluates the actual performance. Furthermore, this study is focused on older adults living in long-term care facilities, a group for which few ADL instruments were tested regarding their psychometric properties.

## 5. Conclusions

This study showed that the Portuguese version of the TIADL had good to excellent test–retest reliability and acceptable measurement precision in older nursing home residents. Furthermore, the results show that the Portuguese TIADL is a valid tool and has high internal consistency. Thus, the TIADL could be a valuable clinical tool for assessing the actual performance of IADLs among Portuguese institutionalised older adults.

## Figures and Tables

**Table 1:** General characteristics of the participants.

Characteristics	All Participants (*n* = 52)	
	Mean (SD)	Min–Max
Age (years)	85.77 (4.2)	(77–95)
Height (cm)	160.0 (8.1)	(149.0–180.7)
Weight (kg)	64.9 (8.0)	(49.6–85.0)
BMI (kg/m^2^)	25.4 (3.0)	(20.4–32.2)
Education (years)	4.7 (2.1)	(3–17)
MMSE (points)	28.7 (1.4)	(22–30)

Note: MMSE, Mini-Mental State Examination; BMI, body mass index.

**Table 2:** Test–retest reliability of the Portuguese TIADL in older nursing home residents.

TIADL Tasks	Mean (SD)					
	Test	Retest	Difference	ICC (95%)	SEM	MDC95
Communication (s)	115.17 (56.16)	119.54 (46.68)	4.37	0.94 (0.89–0.96)	11.55	32.02
Finances (s)	34.19 (11.91)	34.12 (11.87)	−0.07	0.95 (0.92–0.97)	2.63	7.30
Purchases (s)	54.91 (28.56)	53.44 (23.43)	−1.47	0.90 (0.83–0.95)	8.02	22.23
Food (s)	39.67 (11.86)	38.17 (10.87)	−1.50	0.96 (0.93–0.98)	2.26	6.26
Medicine (s)	50.83 (13.57)	51.09 (14.63)	0.27	0.94 (0.90–0.97)	3.42	9.49

Note: TIADL, Timed Instrumental Activities of Daily Living; M, mean; SD, standard deviation; ICC, intraclass correlation coefficient; SEM, standard error of the mean; MDC, minimal detectable change.

**Table 3:** Correlation (Spearman’s rho) results between the Portuguese version of the TIADL, the UFOV test, and semantic and phonemic fluency tests.

*n* = 52	UFOV-PS	UFOV-DA	Semantic Fluency	Phonetic Fluency
Z-score TIADL	0.52 *	0.49 *	−0.47 *	−0.58 *

Note: * correlation is significant at the 0.01 level (2-tailed); *n*, number of participants; UFOV, useful field of view test; PS, processing speed; DA, divided attention.

## Data Availability

The data that support the findings of this study are obtainable from the corresponding author, L.G., upon reasonable request.

## Supplementary Materials

The following supporting information can be downloaded at: https://www.mdpi.com/article/10.3390/geriatrics8060124/s1. Supplementary Materials Portuguese version of the Timed instrumental activities of daily living.
