# Supplementary material for: Translation and Psychometric Properties of the Portuguese Version of the Timed Instrumental Activities of Daily Living (TIADL)
Source: Geriatrics (Basel). 2023 Dec 18;8(6):124. doi: 10.3390/geriatrics8060124 (PMC10743034; doi:10.3390/geriatrics8060124)
Supplement: Supplementary file 1 [file geriatrics-08-00124-s001.zip › geriatrics-2743933-supplementary.pdf]

Supplementary Materials Portuguese version of the Timed instrumental activities of daily living

Tarefas	Materiais	Instruções	Início (I) e Final (F) da contagem de tempo	Pontuação
**Tarefa 1 Comunicação**	Lista telefónica- Páginas brancas 2015-2016	"Vou dar-lhe esta lista telefónica e quero que procure o número do Museu de Évora, o mais rapidamente possível" (nome previamente definido e sempre igual).<br><br>"Percebeu? O que é que tem de procurar?" (Se estiver correto, prossegue-se; se estiver incorreto, repete-se o nome "Museu de Évora"). "Aqui está a lista telefónica, procure o contacto do Museu de Évora, lembre-se quando encontrar o contacto diga-me em voz alta. Pronto! Comece!"	I: A pessoa pega na lista telefónica.<br><br>F: A pessoa assinala com o dedo e/ou refere verbalmente que encontrou.	Tempo total (s) da tarefa
**Tarefa 2 Fazer um troco**	Moedas:<br>3 x 0.20 €<br>4 x 0.10 €<br>5 x 0.05 €<br>4 x 0.01 €	"Você é canhoto ou destro? Agora vou dar-lhe algumas moedas. Quero que coloque 77 cêntimos em cima da mesa o mais rápido que conseguir. Diga-me quando terminar. Quanto é que pedi para colocar em cima da mesa?" (Se estiver correto, prossegue-se; se estiver incorreto, repete-se o valor "77 cêntimos"). "Aqui estão as moedas. Lembre-se, avise-me quando terminar.<br>Pronto! Comece!"<br><br>(Deve anotar-se os erros caso existam).	I: As moedas são colocadas na mão não dominante da pessoa.<br><br>F: A pessoa indica que terminou.	Tempo total (s) da tarefa

**Tarefa 3**<br>**Encontrar produtos numa prateleira**	Uma prateleira de itens variados de mercearia. Medidas (58 cm comprimento, 40 cm largura e 35 cm altura)	"Agora vou mostrar-lhe uma prateleira com vários produtos de mercearia. Eu quero que encontre 2 itens, o mais rápido que conseguir. Os itens são uma lata de atum e uma caixa de fósforos. Mostre-me que encontrou os produtos tocando neles com o dedo. Não tente tirá-los; apenas lhes toque com um dedo. Não importa qual dos dois itens encontrar primeiro." "Quais os itens que deve encontrar?" (Se estiver correto, continue; se estiver incorreto, repete-se os 2 produtos "lata de atum" e "caixa de fósforos"). "Pronto! Comece!"	I: A prateleira é apresentada.<br><br>F: A pessoa toca no 2º item.	Tempo total (s) da tarefa
**Tarefa 4**<br>**Ler os 3 primeiros ingredientes de uma lata de comida**	3 latas de comida (atum, feijão e chocolate em pó);	"Agora vou dar-lhe 3 latas de comida. Eu quero que leia os ingredientes em cada uma das latas. Na lata está escrita a palavra – "ingredientes". Para cada lata, eu quero que leia os 3 primeiros ingredientes, o mais rápido possível. Percebeu? O que é que tem de fazer?" (se correto avançar, se incorreto repetir as instruções) "Aqui está a primeira lata. Leia os primeiros 3 ingredientes o mais rápido possível em voz alta para que eu possa ouvi-lo."	I: A pessoa pega na lata.<br><br>F: A pessoa lê o terceiro ingrediente.	Tempo médio (s) na leitura das três latas.

**Tarefa 5**<br>**Ler as informações em folhetos informativos (bula) de medicamentos**	Dois folhetos informativos, um de paracetamol (genérico) e um de ibuprofeno (genérico).	"1. Vou dar-lhe este folheto informativo de um medicamento, paracetamol, e você tem de encontrar o mais rápido possível e ler a expressão: "como tomar paracetamol". Percebeu? O que é que tem de fazer?" (se correto prosseguir, se incorreto repetir a instrução).<br>"2. Agora vou pedir-lhe para encontrar e ler, novamente o mais rápido possível: "o que é o Ibuprofeno e para que é utilizado". Percebeu, o que é que tem de fazer agora?" (se certo prosseguir, se não repetir a instrução).	I: A pessoa pega no folheto informativo (bula).<br><br>F: A pessoa lê a última palavra da expressão solicitada.	Tempo médio (s) para encontrar e ler as duas expressões
